# A healthy Europe through data, information and cooperation

**DOI:** 10.25646/12947

**Published:** 2024-12-11

**Authors:** Franziska Prütz, Nicole Rosenkötter, Thomas Ziese

**Affiliations:** 1 Robert Koch Institute, Department for Epidemiology and Health Monitoring, Berlin, Germany; 2 Academy of Public Health Services, Düsseldorf, Germany

‘Data for action’ – this motto is inextricably linked with health reporting. This is true in Germany as well as in Europe. Health reporting is a fundamental part of public health. The collection and interpretation of data on the health of the population provides a basis for health policy actions, which can only be based on available and valid data [1, 2].

European Union (EU) health policy focuses on areas such as disease prevention and control, the provision of medicines and health research. The objectives at European level, as at national level, are to protect and improve the health of the population and to ensure that healthcare is adequate and easily accessible. Public health issues such as the prevention of noncommunicable diseases and cancer, immunisation and the fight against antimicrobial resistance play an important role. The relationship is structured in such a way that Member States develop their health policies and are responsible for the organisation and delivery of health services and medical care. The EU supports, coordinates or complements the health policies of its Member States. This is laid down in Article 168 of the Treaty on the Functioning of the EU: ‘Union action, which shall complement national policies, shall be directed towards improving public health, preventing physical and mental illness and diseases, and obviating sources of danger to physical and mental health. Such action shall cover the fight against the major health scourges by promoting research into their causes, their transmission and their prevention, as well as health information and education […]’. In this way, Member States are supported in achieving common goals, pooling resources and tackling common challenges, and funding is made available for health projects across the EU.

The European Health Interview Survey (EHIS) is an important source of data on health in Europe. It is a standardised population survey that was first conducted on a voluntary basis in 2006 – 2009 and has been mandatory for all EU Member States since the second wave (2013 – 2015). The EHIS collects data on health status, health care, health determinants and the socio-economic situation of the population in the EU Member States. The Member States decide on the survey methodology and the details of implementation [3]. The EHIS can be carried out as a stand-alone survey or, as in Germany, embedded in a national health survey. Two articles in this issue of the Journal of Health Monitoring compare Germany and Europe, based on data from EHIS 3 (2018 – 2020).

In the first article, Baumert et al. report on the prevalence of three disease groups of public health concern in Germany – diabetes mellitus, cardiovascular diseases and chronic respiratory diseases – and place them in a European context. The prevalence of these diseases is higher in Germany than the European average. However, the proportion of people with the diseases who rate their health as very good or good is also higher in Germany.

The article by Krause et al. focuses on the utilisation of healthcare: The results show that the use of all health services – outpatient and inpatient care, medical examinations and the use of medicines – is higher in Germany than in the European average. Demographic and social differences, however, are similar in Germany and at European level.

Common health indicators are an essential basis for international comparisons. The European Core Health Indicators (ECHI) have provided the EU with a coordinated set of indicators since the late 1990s. The article by Tijhuis et al. discusses the sustainability of the ECHI indicator set and considers ways to improve the future use of the indicators. The authors argue that this requires sustainable funding and governance as well as a permanent structure.

The fourth article in this thematic issue also argues for permanent structures. Thißen et al. describe the three European projects BRIDGE Health, InfAct and PHIRI, which are building on each other towards a European health information system. Their results include the establishment of a health information portal, the development of guidelines and training courses, and the implementation of a federated research infrastructure. However, to achieve the goal of a European health information system, these projects also require long-term funding.

The four articles present examples of perspectives on health reporting in Europe, from data collection, data analysis, interpretation, formulation of needs for action and the actual reporting, to the development of indicator sets and the provision of health data and information in a health information system. Why do European countries differ in the prevalence of diseases and in the use of health services? Which sets of indicators are suitable for mapping the health situation, healthy lifestyles or social inequalities in Europe? How can this information be presented in a way that is easily accessible and target group-appropriate? Europe-wide information on population health can be used to develop national strategies to address health policy challenges. It can also support the search for best practice examples of policies and interventions in European countries, provided that the relevant structures are continuously developed and reliably available. Overall, it seems that health (and health reporting) needs much more Europe – in order to facilitate productive use of data, experiences, but also regional differences.
